# A Model-Based Estimate of Regional Wheat Yield Gaps and Water Use Efficiency in Main Winter Wheat Production Regions of China

**DOI:** 10.1038/s41598-017-06312-x

**Published:** 2017-07-20

**Authors:** Zunfu Lv, Xiaojun Liu, Weixing Cao, Yan Zhu

**Affiliations:** 1 National Engineering and Technology Center for Information Agriculture, Key Laboratory for Crop System and Decision Making, Ministry of Agriculture, Jiangsu Key Laboratory for Information Agriculture, Nanjing Agricultural University, 1 Weigang Road, Nanjing, Jiangsu 210095 P. R. China; 2 Department of Agronomy, The Key Laboratory for Quality Improvement of Agricultural Products of Zhejiang Province, College of Agriculture and Food Science, Zhejiang A & F University, Lin’an, Hangzhou, 311300 P. R. China

## Abstract

Wheat production is of great importance for national food security and is greatly influenced by the spatial variation of climatic variables, soils, cultivars, etc. This study used WheatGrow and CERES-Wheat models integrated with a GIS to estimate winter wheat productivity, yield gap and water use in the main wheat production regions of China. The results showed that the potential wheat yield gradually increased from south to north and from west to east, with a spatial distribution consistent with the accumulated hours of sunshine. The gap between potential and actual yield varied from 382 to 7515 kg ha^−1^, with the highest values in Shanxi, Gansu and Shaanxi provinces and the lowest values in Sichuan province. The rainfed yield decreased gradually from south to north, roughly following the pattern of the ratio of accumulated precipitation to accumulated potential evapotranspiration. Under the scenario of autoirrigation, relatively high irrigation water use efficiency was found in western Shandong and southern Sichuan, as well as in northern Henan, Shanxi and Shaanxi. Furthermore, the limiting factors were analysed, and effective measures were suggested for improving regional winter wheat productivity. These results can be helpful for national policy making and water redistribution for agricultural production in China.

## Introduction

Wheat is the second major food crop in China. In 2010, wheat was grown on approximately 24.3 million hectares, comprising 22.1% of the total area of China’s grain crops and 21.1% of the total grain crop production^1^. Prediction of wheat productivity and identification of the factors limiting regional wheat production have great significance for improving potential wheat productivity and setting agricultural production policy. Crop simulation models are useful tools for estimating regional crop productivity. The methods of model-based estimation of regional crop productivity generally fall into two groups: semi-empirical models and mechanistic models. Semi-empirical models are built on the statistical relationships between crop yield or phenology and environmental variables^2^, and they mainly consider environmental variables such as light, temperature and water^3, 4^. These types of model do not consider the mechanism of crop growth and development^5^. In contrast, mechanistic models are based on the processes of crop growth and yield formation, and they can thus predict the growth and development dynamics of a given variety under different pedoclimatic conditions^6^. Mechanistic crop models, such as DSSAT, APSIM and WheatGrow, have been used extensively to evaluate the effects of environmental, biological, and management changes on crop growth and production^7–12^.

At present, assessment of the impact of climate change on crop productivity using mechanistic crop models at the regional scale are mainly based on the results of several representative agricultural eco-sites^13, 14^. Although it is easy to implement such model and less time consuming to obtain the simulation output, such predictions ignore the complex spatial heterogeneity of weather, soil, cultivars and managements, and thus often generate simulation errors^2^. Therefore, the evaluation results from only a few eco-sites can not represent the characteristics of the whole region.

By integrating a process-based growth model with Geographic Information System (GIS), various studies have evaluated the performance of regional simulations using site-scale crop models^15–17^. However, only a few studies have considered regional simulation of crop productivity in China because of the scarcity of input data^18^, although the WheatGrow model was scaled up from site to regional levels by integration with GIS^19^. Xiong simulated China’s potential maize production at a regional scale using the CERES-Maize model^20^. Wu quantified production potentials of winter wheat in the North China Plain by combining the WOFOST model and GIS^21^. In addition, regional crop models have also been integrated with other models including regional climate models to examine the effects of climate changes^6^. Although these studies have generally explored regional productivity, some details in the spatial inputs of weather, cultivars, soil and management have been simplified. Recent studies have identified numerous scientific problems associated with the data sources and methods used in regional potential yield studies^22–24^, such as: poor quality of weather and soil data, unrealistic assumptions about the cropping-system context and poorly calibrated crop simulation models. Potential yield estimates with coarse-scale data sources can seriously distort predictive results^23^. Therefore, the productivity and yield gaps of winter wheat should be quantified with a larger and higher spatial resolution compared with previous studies^21–25^.

Water is an important limiting factor for wheat growth in the wheat-producing regions in China. On the one hand, China needs enough wheat productivity to feed the increasing population^25^. On the other hand, due to the lack of surface water in northern China, groundwater has become the main source of irrigation water^26^. To meet the irrigation requirement, groundwater has been over-pumped^27^. The overuse of water resources has decreased agricultural sustainability and caused serious environmental hazards^26^. Prediction of regional wheat productivity and water use efficiency in the main wheat production regions of China could help us identify a balance between wheat yield and water use at the regional scale.

The primary objectives of this study were (1) to estimate the regional productivities of winter wheat at different levels in the main production regions of China and to identify the spatial variations in wheat yield caused by climate variability; (2) to reveal the yield gap between potential yield and actual yield, and the degree to which wheat is limited by water in the main wheat production regions of China; and (3) to analyse irrigation water use efficiency in the main wheat production regions of China. The results should be useful for strategy development and policy making for regional wheat production and water redistribution.

## Methods

### Study region

**Figure 1:**
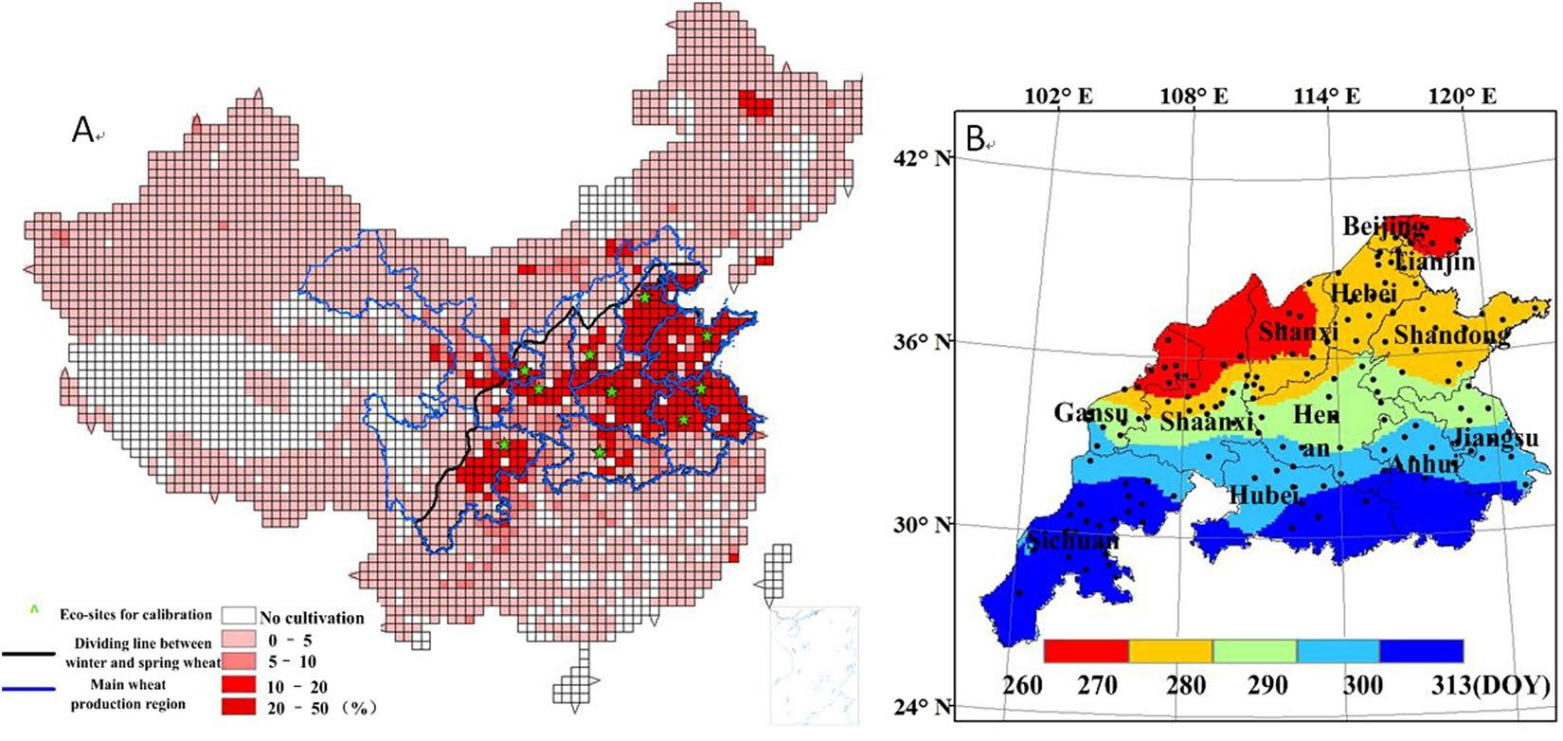
(**A**) Distribution map^9^ of the wheat planting area proportion, main wheat growing region and dividing line between winter and spring wheat (black line); (**B**) Julian day of average sowing date from 1998 to 2003 (This figure was created by ArcGIS 9.3, http://www.esri.com/arcgis/).

The ten major production provinces with the largest relative wheat sowing fractions were chosen as study provinces, These included Shandong, Jiangsu, Hubei, Henan, Shanxi, Shaanxi, Gansu, Hebei, Anhui and Sichuan provinces, as shown inside the blue line in Fig. 1A. Spring wheat is planted in the areas above the black line, while winter wheat is planted in the areas below the black line^28^. Figure 1B shows the average winter wheat sowing dates (Day of Year, DOY) during the period from 1998 to 2003 in the study regions^9^.

### Meteorological data

The meteorological data from 656 weather stations in China from 1998 to 2008, including daily maximum temperature (°C), minimum temperature (°C), precipitation (mm) and hours of sunshine (hours), were provided by the China Meteorological Administration (http://data.cma.cn). Solar radiation data were converted from hours of sunshine by the method of Pohlert^29^. The raw DEM (Digital Elevation Model) data were provided by the Consultative Group on International Agricultural Research^30^. The raster data at a resolution of 0.1° × 0.1° were generated using ArcGIS 9.3 software.

Daily meteorological data from 1998 to 2008 at 656 weather stations in China were interpolated with the thin-plate smoothing splines method in the ANUSPLIN software package version 4.3, to produce high-resolution (0.1° per grid) daily meteorological data sets^30–35^.

### Soil data

The soil data were mainly derived from the Harmonized World Soil Database (HWSD)^36^. The spatial resolution of the HWSD is 30 arc-seconds. Soil textural data for two layers, 0–30 cm and 30–100 cm, were included in the HWSD. The spatial soil information from the HWSD provided most of the soil parameters required by the crop models within a 100-cm deep soil profile. The HWSD data were derived from the digital soil map of China^37^ at a scale of 1:1 000 000, which provided most of the soil parameters (bulk density, organic carbon, pH, water storage capacity, soil depth, cation exchange capacity of the soil, the clay fractions, total exchangeable nutrients, lime and gypsum contents, sodium exchange percentage, salinity, saturated moisture content, field capacity, wilting point, textural class and granulometry) for crop models. The three soil parameters (saturated moisture content, field capacity and wilting point,) required were estimated from soil texture and organic matter^38^. We assumed that the maximum rooting depth of wheat in this study was 100 cm^39^. Soil moisture were obtained from the China Land Soil Moisture Data Assimilation System (CLSMDAS) based on the Ensemble Kalman Filter and land process models—Community Land Model Version 3.0 (CLM3.0), which were developed by the US National Center for Atmospheric Research (NCAR)^40^.

The raw soil data from the HWSD were converted into regional vector data according to the soil types (FAO90 soil classification system). Through the overlay of the interpolated meteorological raster layer and soil vector layer, the soil type with the maximum area in each grid was selected to represent the soil type of each grid. Finally, the corresponding soil physical and chemical properties in every grid of the study region were obtained from the soil type attribute database.

### Cropping management

WheatGrow and CERES-Wheat assume that weeds, diseases, and pests are controlled and fertilizers are optimally available. Irrigated and rainfed conditions are known to affect crop yield differently^41^; thus, both are considered in the present study. Potential yield is simulated based on the irrigation method of automatic irrigation, and rainfed yield is simulated based on rainfed conditions. In automatic irrigation, the optimal amount of water is provided to compensate for the estimated soil water deficiency. During the simulation, irrigation was applied when soil moisture was lower than 65% of field capacity. This implies that water was always available when needed. In rainfed conditions, no irrigation was supplied during the simulation. Under rainfed conditions, crops may incur a certain degree of water-stress during drought conditions.

**Figure 2:**
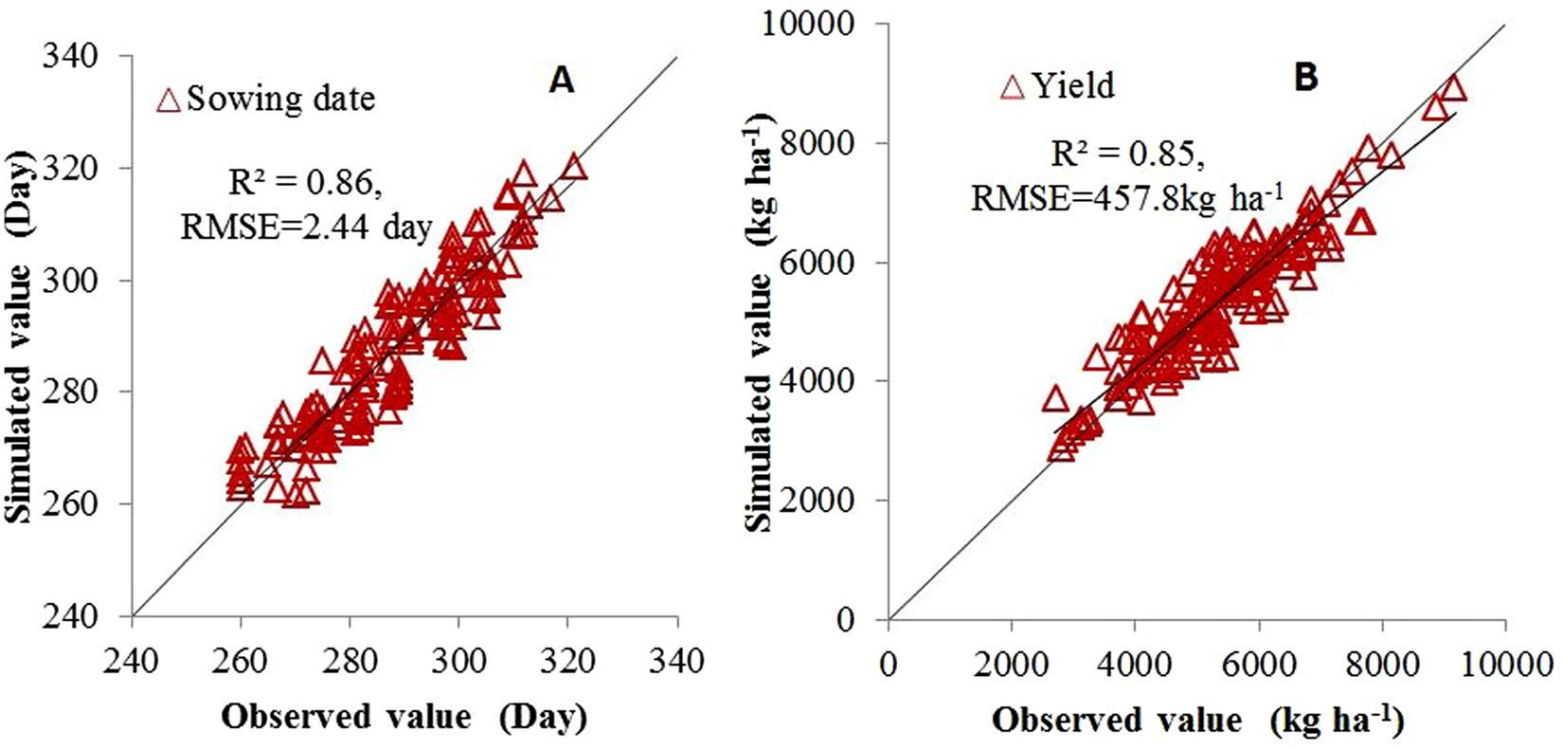
Validation of the interpolation method for wheat sowing dates and actual yield from 1998 to 2008.

A second-order spline using latitude and longitude as independent variables was fitted to interpolate the surfaces of sowing date with a resolution of 0.1° × 0.1° ^9^. A third-order spline using latitude, longitude and altitude as independent variables was fitted to interpolate the surfaces of actual yield with a resolution of 0.1° × 0.1°. The actual yield data in each province were used to generate the yield surface of their respective province. Then, the yield surfaces of all provinces were combined to generate the yield surface of the main wheat production region. Wheat sowing dates and actual yields at 120 eco-sites (Fig. 1B) were used to generate the surfaces of wheat sowing dates and actual yields from 1998 to 2008. Wheat sowing dates and actual yields at the 28 other eco-sites were used to validate regional wheat sowing dates and actual yields. The coefficient of determination (r^2^) and root mean square error (RMSE) for the sowing date were 0.86 and 2.44 days, respectively (Fig. 2A), and r^2^ and RMSE values for actual yield were 0.85 and 457.8 kg ha^−1^, respectively (Fig. 2B). Cropping density for all grids was the same as that in the neighbouring ecosite (Fig. 1B). We did not consider crop rotation in this research. The soil conditions were reset annually. The beginning of the simulation was ten days before sowing. The initial soil moisture was set to 65% of field capacity.

### WheatGrow and CERES-Wheat models

WheatGrow is composed of six submodels: photosynthesis and biomass production^42^; dry matter partitioning^43^; apical development and phenological development^44–46^; organ growth and yield formation^47, 48^; soil water balance^49, 50^; and nitrogen (N), phosphorus (P) and potassium (K) dynamics^51^. Several studies have used the WheatGrow model at site or regional scales^52, 53^, and the results showed that the model was able to simulate the effects of climate factors and irrigation regimes on wheat growth and yield formation under different production systems.

The CERES-Wheat model distributed with DSSAT v4.6^54^ is a dynamic mechanistic crop simulation model that calculates phenological development and growth processes in response to environmental and management factors. To simulate crop growth in daily time steps, the model considers cultivars, weather, soil and planting conditions. Development and growth processes are influenced by water and N balance submodels^55^.

### Model calibration and validation

A total of 148 historical observations of wheat phenology (sowing, heading and maturity dates), grain yield and management practices from 1998 to 2008 were obtained from the National Meteorological Center Library of China. Ten representative eco-sites (green stars in Fig. 1A) were selected, Weifang, Shijiazhuang, Zhengzhou, Huai’an, Hefei, Linfen, Wugong, Xifeng, Bazhong and Zhongxiang, which were from the provinces of Shandong, Hebei, Henan, Jiangsu, Anhui, Shanxi, Shaanxi, Gansu, Sichuan and Hubei, respectively.

Based on the grain yield and the dates of jointing, heading and maturity from 1998 to 2003 at the ten representative eco-sites, the region-specific cultivar parameters were estimated with two models for the previously mentioned provinces. A Markov Chain Monte Carlo (MCMC)-based method was used for parameter estimation of WheatGrow and CERES-Wheat^56^. One optimal set of parameters was chosen for each province (Table 1).

**Table 1:** Regional cultivar parameter values used for the WheatGrow and the CERES-Wheat models.

Provinces	WheatGrow										CERES-Wheat						
	PVT(day)	IE	PS	TS	FDF	SLA	LT	GW(g)	HI	TA	P1V	P1D	P5	G1	G2	G3	PHINT
Anhui	30	1.33	0.0060	0.69	0.79	0.0028	46.3	37.02	0.4	0.52	33.8	52	420	29	38	1.8	80
Hebei	37	1.61	0.0068	0.84	0.69	0.0027	63.31	40.50	0.38	0.61	36.2	76	436	29	39	2.1	90
Jiangsu	25	1.68	0.0062	0.67	0.57	0.0026	48.32	44.01	0.38	0.65	32.6	53	461	32	42	1.9	80
Henan	33	1.46	0.0055	1.01	0.82	0.0031	52.27	39.69	0.37	0.46	37.0	36	413	31	41	1.8	80
Gansu	39	1.41	0.0054	0.81	0.69	0.0026	71.94	39.76	0.44	0.47	36.9	80	397	25	36	1.8	95
Shandong	40	1.50	0.0006	0.86	0.74	0.0031	63.69	42.97	0.41	0.54	33.8	48	406	34	45	2.1	85
Hubei	24	1.46	0.0053	1.05	0.70	0.0027	41.42	42.66	0.37	0.63	30.5	54	405	25	36	1.5	70
Sichuan	16	1.03	0.0036	0.40	0.54	0.0024	46.6	37.3	0.39	0.55	26.1	32	442	20	30	1.3	60
Shanxi	37	1.65	0.0072	1.24	0.68	0.0026	82.43	47.06	0.38	0.54	36.5	76	415	28	37	1.7	90
Shaanxi	38	1.64	0.0069	0.96	0.66	0.0032	47.64	31.52	0.36	0.45	41.0	43	422	27	37	1.8	90

PVT: Physiological vernalization time (d).

IE: Intrinsic earliness.

TS: Temperature sensitivity.

PS: Photoperiod sensitivity.

FDF: Filling duration factor.

SLA: Specific leaf area under optimum conditions (ha kg^-1^).

LT: The thermal time between two successive leaf tip appearances (°C∙d).

HI: Harvest index.

GW: 1000-grain weight (g).

TA: Tilling ability

P1V: Days required to complete vernalization.

P1D: Percentage reduction in development rate in a photoperiod.

P5: Grain filling phase duration (°C∙d).

G1: Kernel number per unit canopy weight at anthesis (g).

G2: Standard kernel size under optimum conditions (mg).

G3: Standard non-stressed dry weight of a single tiller at maturity (g).

PHINT: Interval between successive leaf tip appearances (°C∙d).

Field observations (jointing, heading, maturity date and grain yield) for two growing seasons from 2003 to 2005 at the 148 eco-sites were used for model validation at the regional scale. Daily meteorological data were selected for each eco-site. The soil data including soil physical and chemical properties, were selected according to the latitude and longitude of eco-sites using GIS. The calibrated regional cultivar parameters at the 10 eco-sites above represent the ecotype cultivars of the ten provinces. Cropping management was consistent with the actual situation in the field. The fitness between observed and simulated values was assessed with the coefficient of determination (r^2^) and root mean square error (RMSE) (equation (1)).

1 $${\rm{RMSE}}=\sqrt{\frac{{\sum }_{{\rm{j}}=1}^{{\rm{N}}}{({{\rm{O}}}_{{\rm{j}}}-{{\rm{S}}}_{{\rm{j}}})}^{2}}{{\rm{N}}}}$$

where O_j_ and S_j_ were the observed and estimated values, respectively; N was the total number of samples.

### Simulation of regional wheat productivity

By combining WheatGrow and CERES-Wheat models with GIS, the crop models were scaled up from site to regional levels. The spatial data in the study region were prepared and processed in advance by interpolation and overlaying with the aid of GIS and were divided into homogeneous grids. The grid is taken as the basic simulation unit, and each grid has a complete set of input data (weather, soil, cultivar and management). Regional productivity was simulated with the WheatGrow and CERES-Wheat model for each grid cell. Regional simulation was performed for a total of 14,212 grid cells in the main wheat production regions for the period from 1998 to 2008. Spatial distributions of the maturity date, potential yield (Yp), rainfed yield (Yr), yield gap (Yg) between potential and actual yield, water limitation index (WLI), average irrigation amount and irrigation water use efficiency (IWUE) were the average values of regional simulations from 1998 to 2008.

The actual wheat yields (Ya) at 148 eco-sites (Fig. 1B) were used to interpolate the surfaces of actual yields. A second-order spline using latitude and longitude as independent variables was fitted to interpolate the surfaces of actual yields with a resolution of 0.1° × 0.1°.

The yield gap (Yg) was defined as the gap between potential and actual yields^57, 58^ (equation (2)), and the yield gap percentage was defined as the Yg relative to the potential yields, expressed as a percentage (equation (3)).

2 $${\rm{Yg}}={\rm{Yp}}-{\rm{Ya}}$$

3 $${\rm{Yg}}\,{\rm{percentage}}={\rm{Yg}}/{\rm{Yp}}\,\ast \,100 \% $$

The gap between Yp and Yr, called the ‘water limitation index’ (WLI) (equation (4)), provides a measure of the degree to which crops are limited by water^59^.

4 $${\rm{WLI}}={\rm{Yp}}-{\rm{Yr}}$$

Irrigation water use efficiency can be expressed as IWUE^60^ (equation (5)), which can be defined as follows:

5 $${\rm{IWUE}}=({\rm{Yp}}-{\rm{Yr}})/{\rm{I}}$$

where Yp was the potential production (kg ha^−1^), Yr was the rain-fed production (kg ha^−1^) and I was the irrigation amount (mm) in an automatic irrigation scenario.

The root mean square deviation (RMSD)^61^ at the regional scale was used to evaluate the uncertainty for estimating maturity date, potential yield and rainfed yield. RMSD represented the dispersion among estimated values. The RMSD can be calculated according to the equations (6,7).

6 $$\bar{{\rm{S}}}=\frac{1}{{\rm{K}}}{\sum }_{{\rm{i}}}^{{\rm{K}}}{{\rm{S}}}_{{\rm{i}}}$$

7 $${\rm{RMSD}}=\sqrt{\frac{{\sum }_{i=1}^{K}{({{\rm{S}}}_{{\bf{i}}}-\bar{{\rm{S}}})}^{2}}{{\rm{K}}}}$$

where K was the number of years; $\bar{{\rm{S}}}$ was the average estimated value from 1998 to 2008; S_i_ was the estimated value in the *i* year, and RMSD was root mean square deviation.

## Results

### Model validation

**Figure 3:**
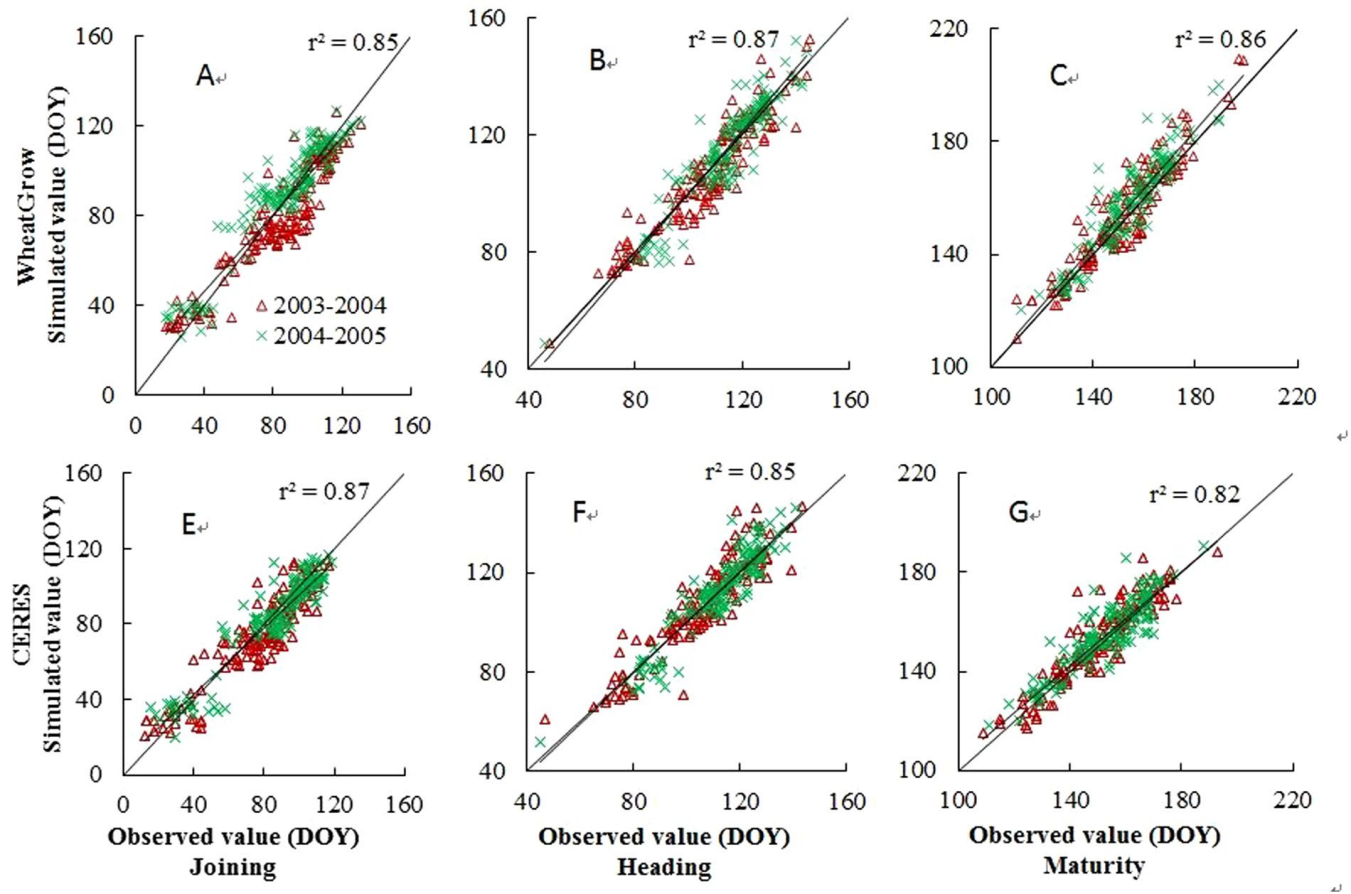
Comparison between simulated and observed values of jointing, heading and maturity dates in wheat at regional scale with the WheatGrow (**A**,**B** and **C**) and the CERES-Wheat (**E**,**F** and **G**) models.

**Figure 4:**
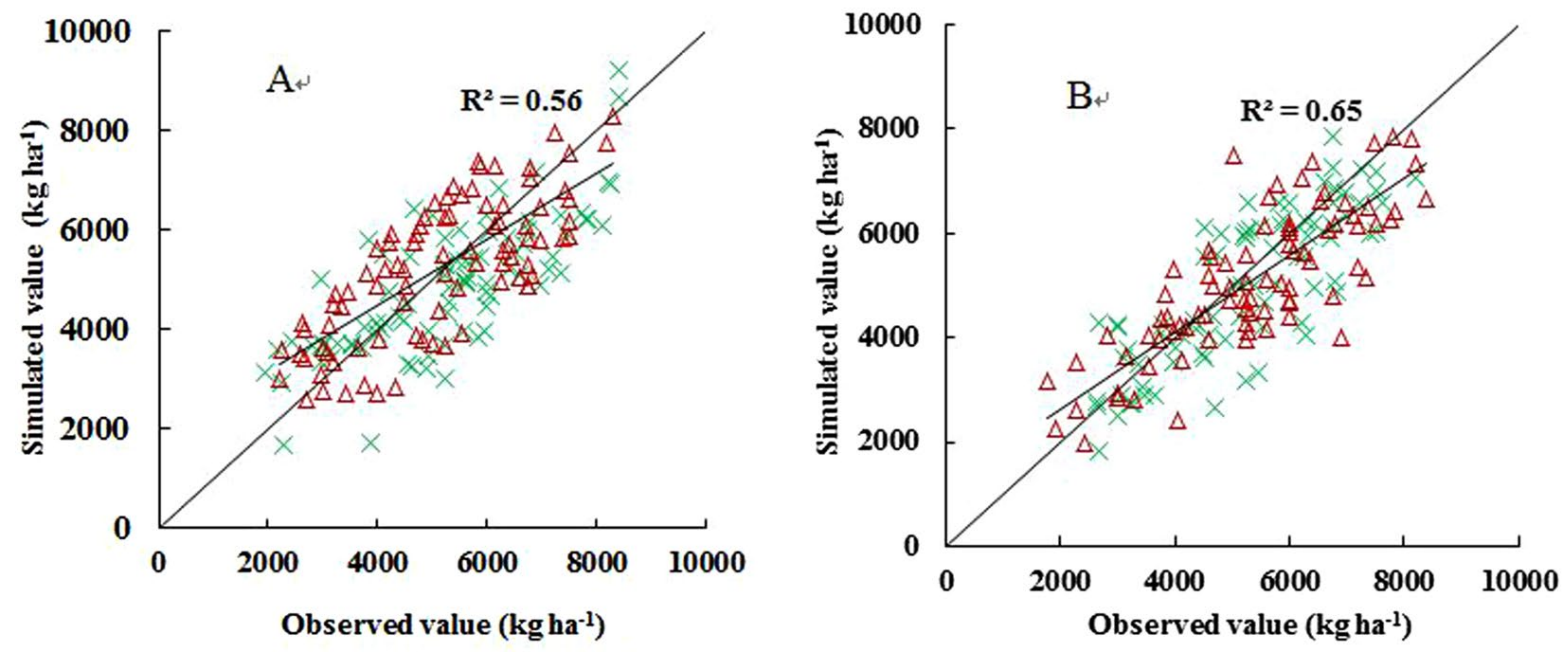
Comparison between simulated and observed values of grain yield in wheat at the regional scale with the CERES-Wheat (**A**) and WheatGrow (**B**) models.

Field observations (jointing date, heading date, maturity date and grain yield) for two growing seasons from 2003 to 2005 at the above mentioned 148 eco-sites were used for model validation at the regional scale. Figure 3 shows that r^2^ values between estimated and observed values of jointing date, heading date and maturity date were 0.87, 0.85 and 0.82 (CERES); 0.85, 0.87 and 0.86 (WheatGrow), respectively. RMSE values between estimated and observed values of jointing date, heading date and maturity date were 9.4, 7.8 and 6.6 day (CERES); 9.6, 7.2 and 6.3 day (WheatGrow), respectively. Figure 4 shows that r^2^ values between the estimated and observed grain yields are 0.56 and 0.65, and RMSEs are 1034 kg ha^−1^ and 965 kg ha^−1^ for CERES-Wheat and WheatGrow, respectively.

### Spatial variability of meteorological elements

**Figure 5:**
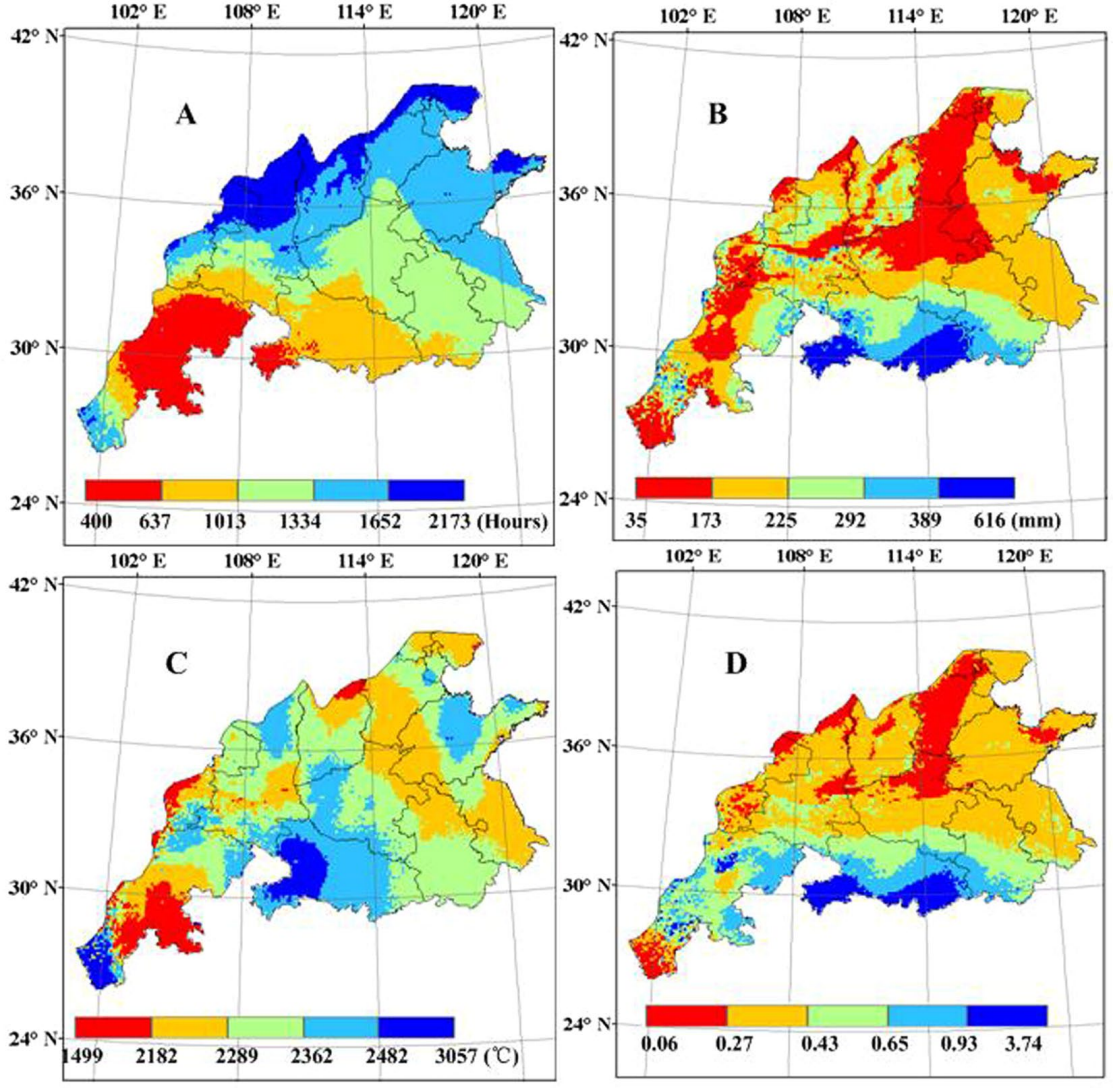
Spatial distribution of meteorological factors in the main winter wheat production regions of China from 1998 to 2008 (**A**) accumulated hours of sunshine (**B**) accumulated precipitation (**C**) accumulated effective temperature (**D**) accumulated the ratio of precipitation to potential evapotranspiration (P/PET) (This figure was created by ArcGIS 9.3, http://www.esri.com/arcgis/).

Analyses were made on the spatial variability of accumulated hours of sunshine, precipitation, effective temperature and the ratio of precipitation to potential evapotranspiration (P/PET) during the winter wheat growing season in the main wheat production regions of China (Fig. 5). The results indicated that the accumulated hours of sunshine during the wheat growing period gradually increased with increasing longitude and latitude. The fewest accumulated hours of sunshine were found in Sichuan province, while the most accumulated hours of sunshine were found in the north of Shanxi province. Accumulated precipitation during the wheat growing period increased from less than 35 mm in the north to 616 mm in the south of the study region. The highest accumulated precipitation was located in Hubei province. Since changes in dry/wet status were very pertinent to crop yield, the change of dry/wet status in the growing season were analysed using the ratio of precipitation to potential evapotranspiration (P/PET). PET was calculated using the Priestley-Taylor method^62^. The spatial pattern of accumulated P/PET was similar to that of cumulative precipitation. However, in Sichuan province, relatively high accumulated P/PET and relatively low accumulated precipitation were observed. The highest accumulated effective temperature was found in the west of Hubei province, which dependent on both the length of wheat growing period and average temperature during wheat growing period. In contrast, along the same latitude, the lowest accumulated effective temperature was located in Sichuan province.

### Spatial variability of maturity date

**Figure 6:**
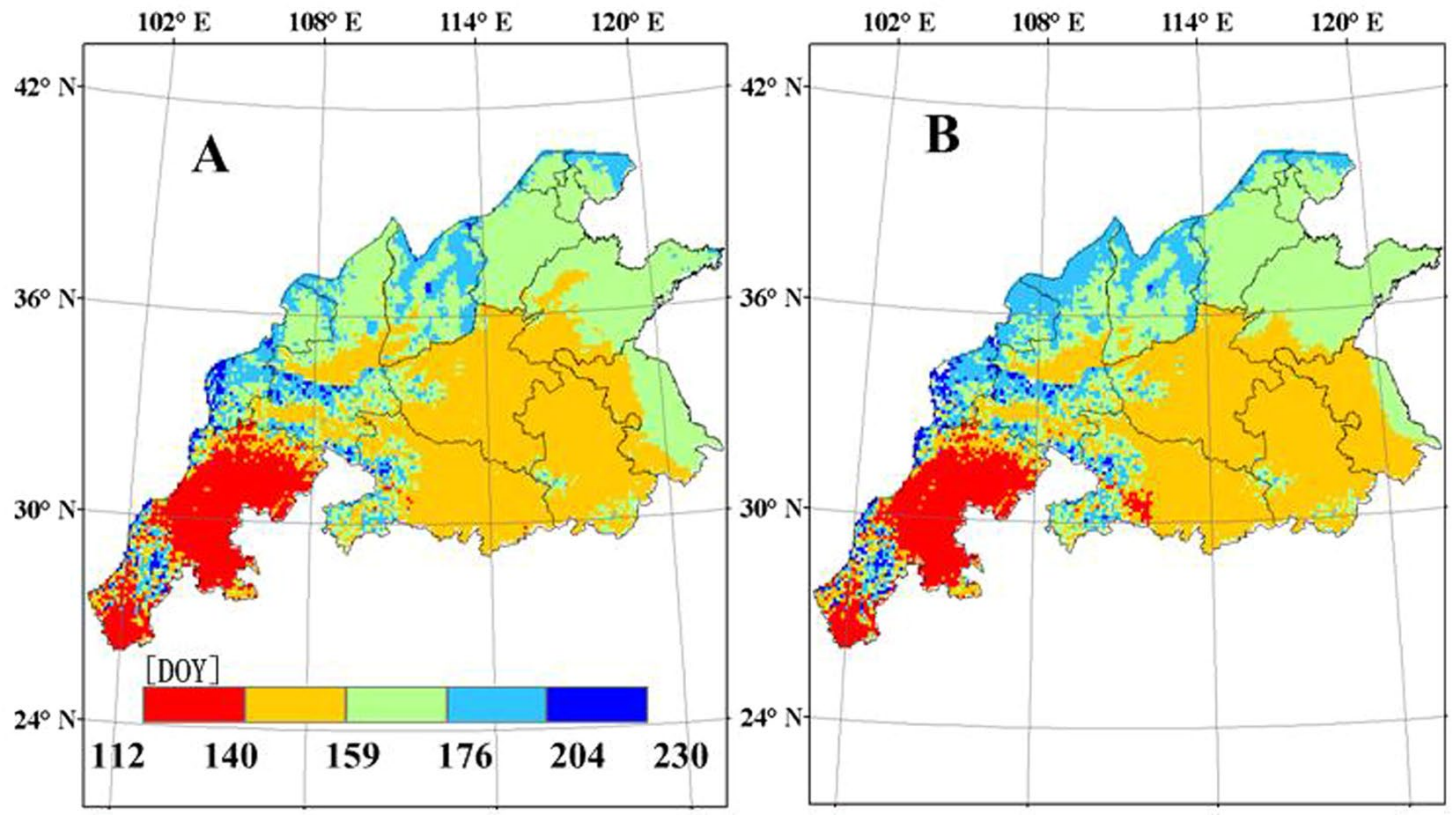
Spatial distribution of simulated maturity (DOY) of winter wheat in the main production regions of China from 1998 to 2008, (**A**) CERES-Wheat; (**B**) WheatGrow (This figure was created by ArcGIS 9.3, http://www.esri.com/arcgis/).

There is a gradient of increasing maturity from the north to the south and from the east to the west in the main production regions (Fig. 6). The earliest maturity date was in Sichuan province, followed by Hubei province, whereas the latest maturity date was in the northern parts of Gansu, Shanxi, Shaanxi and Hebei provinces. The maturity was mainly affected by the temperature for similar varieties of winter wheat. Therefore, the estimated maturity was later in regions at higher latitudes than in those at lower latitudes, in regions with higher elevation than in those with lower elevation and regions near the coast than in those far from the coast, due to lower temperatures in the former regions.

### Potential yield

**Figure 7:**
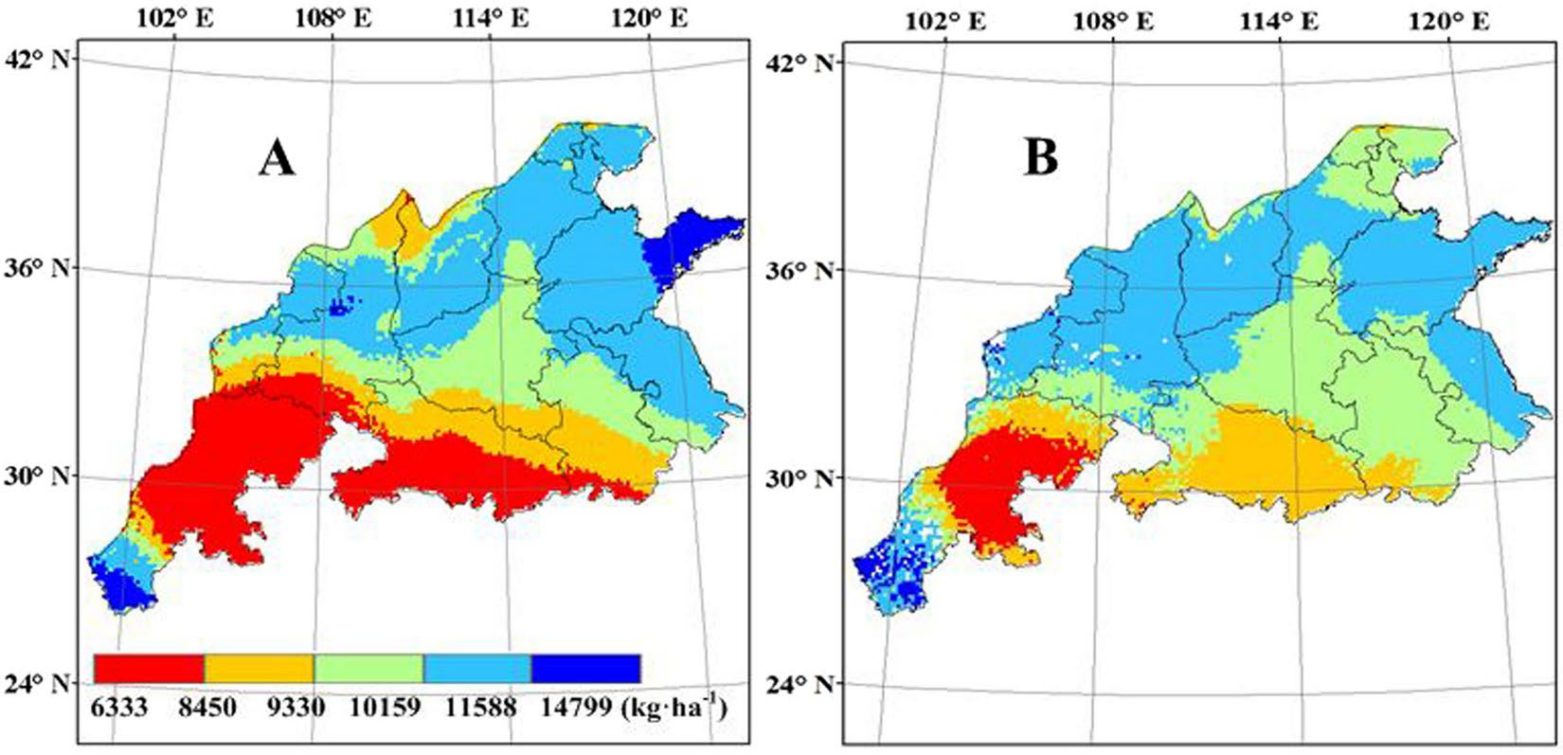
Spatial distribution of average potential yield of winter wheat from 1998 to 2008 in the main production regions of China, (**A**) CERES-Wheat; (**B**) WheatGrow (This figure was created by ArcGIS 9.3, http://www.esri.com/arcgis/).

Potential yield varied from 6333 to 14799 kg ha^−1^ in the main wheat production regions (Fig. 7), and decreased from north to south. Wheat potential yield in the eastern regions near the coast is larger than that in the western regions. Along the same latitude, potential yields increased from west to east. The spatial pattern of potential yield simulated by CERES-Wheat was quite similar to the yield simulated with WheatGrow. Shandong province has the highest potential yield (10159–14799 kg ha^−1^), due to its long growing season and high solar radiation. The potential yields simulated by CERES-Wheat reach 11588 kg ha^−1^ in the eastern areas of Shandong province and are higher than those simulated by WheatGrow. However, in Hubei province, the potential yields simulated by WheatGrow reach 8450 kg ha^−1^, higher than those simulated by CERES-Wheat.

### Yield gap between potential yield and actual yield

**Figure 8:**
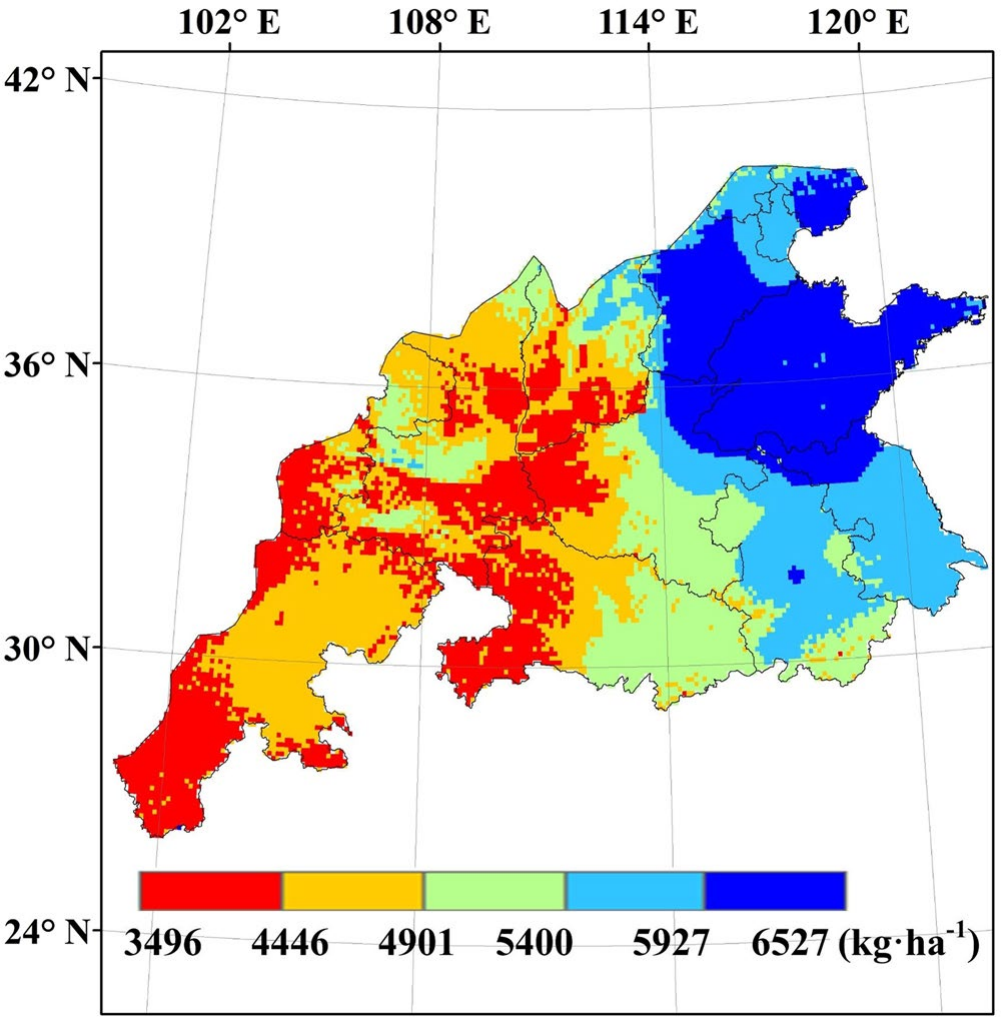
Spatial distribution of average actual yield of winter wheat from 1998 to 2008 in the main production regions of China (This figure was created by ArcGIS 9.3, http://www.esri.com/arcgis/).

**Figure 9:**
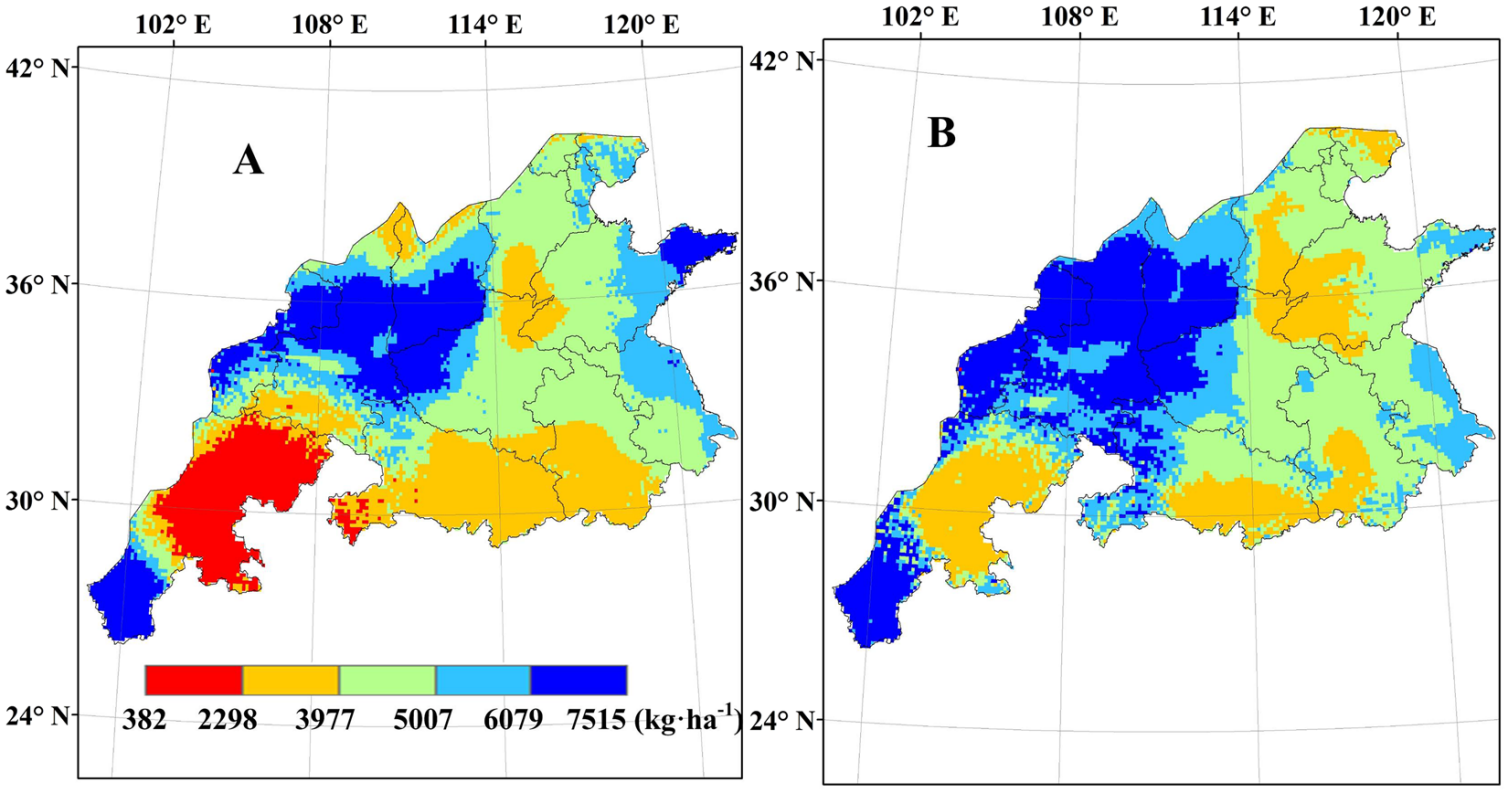
Average yield gap (Yg) percentage from 1998 to 2008 in the main winter wheat production regions of China, (**A**) CERES-Wheat; (**B**) WheatGrow (This figure was created by ArcGIS 9.3, http://www.esri.com/arcgis/).

The yield gap between the potential yield and the actual yield (Fig. 8) achieved by farmers, represents the exploitable yield gap. The yield gaps vary from 382 to 7515 kg ha^−1^, with the highest values in Shanxi, Gansu and Shaanxi provinces and the lowest values in Sichuan province (Fig. 9). The yield gaps simulated by CERES-Wheat model were lower than 2977 kg ha^−1^ in the most regions of Sichuan province, while the yield gaps simulated by WheatGrow model were lower than 4631 kg ha^−1^ in the most regions of Sichuan province. The yield gaps simulated by CERES-Wheat model were higher than 6245 kg ha^−1^ for the east regions of Shandong provinces, while the yield gaps simulated by WheatGrow model were between 4631 kg ha^−1^ and 5336 kg ha^−1^ in the east regions of Shandong provinces.

### Rainfed yield and WLI

**Figure 10:**
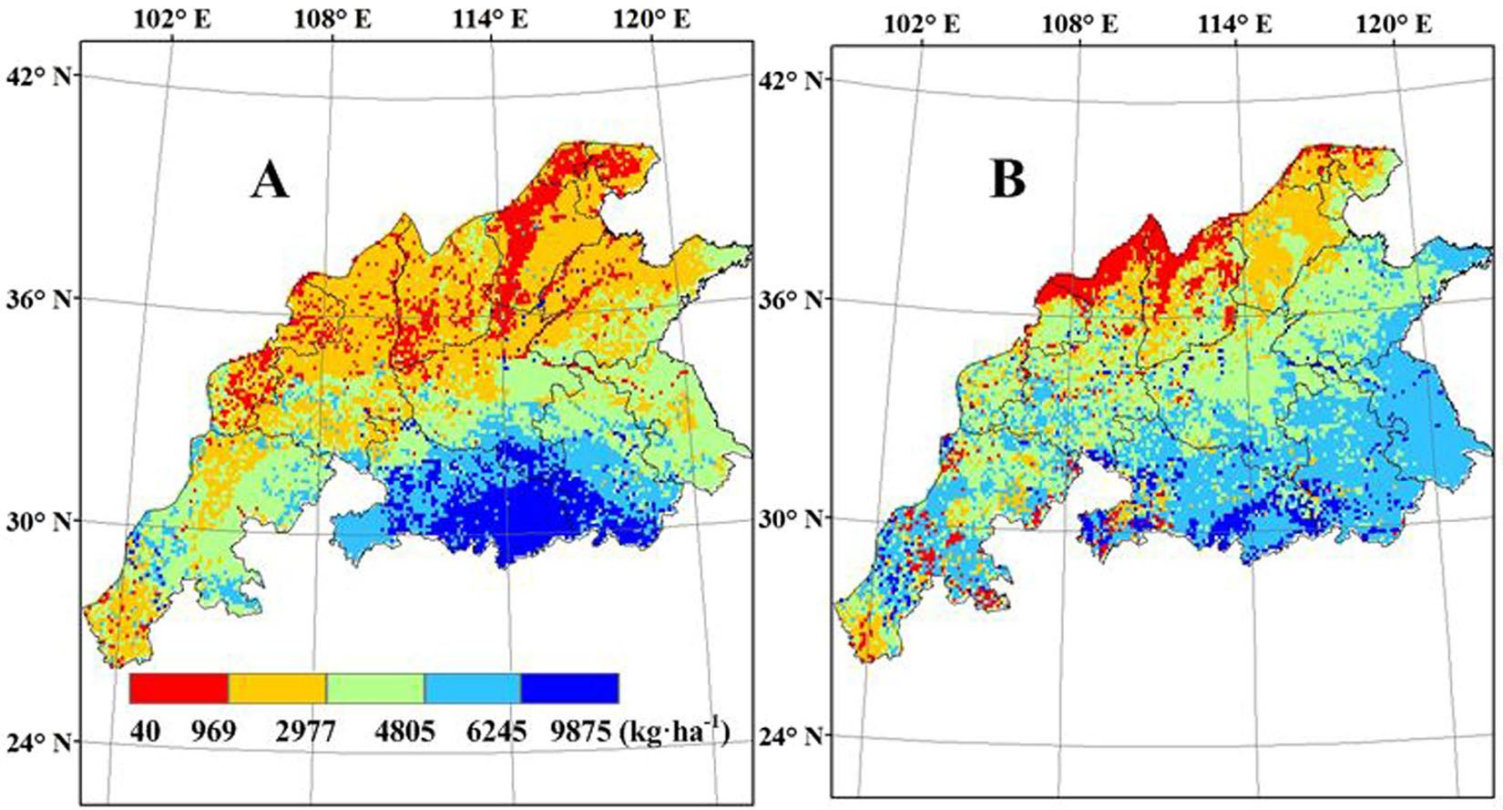
Spatial distribution of average simulated rainfed yield of winter wheat from 1998 to 2008 in the main production regions of China, (**A**) CERES-Wheat; (**B**) WheatGrow (This figure was created by ArcGIS 9.3, http://www.esri.com/arcgis/).

Rainfed yield varied between 40 and 9875 kg ha^−1^ (Fig. 10). The spatial patterns of rainfed yield simulated by WheatGrow agree well with those simulated by CERES-Wheat. Rainfed yield increased from the northern to the southern region, roughly following the trend of accumulated P/PET. The results reveal good linear relationships between simulated rainfed yield and accumulated P/PET, with correlation coefficients of 0.58 and 0.72 for WheatGrow and CERES-Wheat, respectively. The highest rainfed yields simulated by the two models were located in Hubei and Anhui provinces. Rainfed yields simulated by the WheatGrow model are higher than those simulated by the CERES-Wheat model in Shandong and Jiangsu provinces. Compared with the rainfed yields simulated by WheatGrow, the higher rainfed yields simulated by CERES-Wheat were in the regions with higher accumulated precipitation, while the lower rainfed yields simulated by CERES-Wheat were in the regions with lower accumulated precipitation.

**Figure 11:**
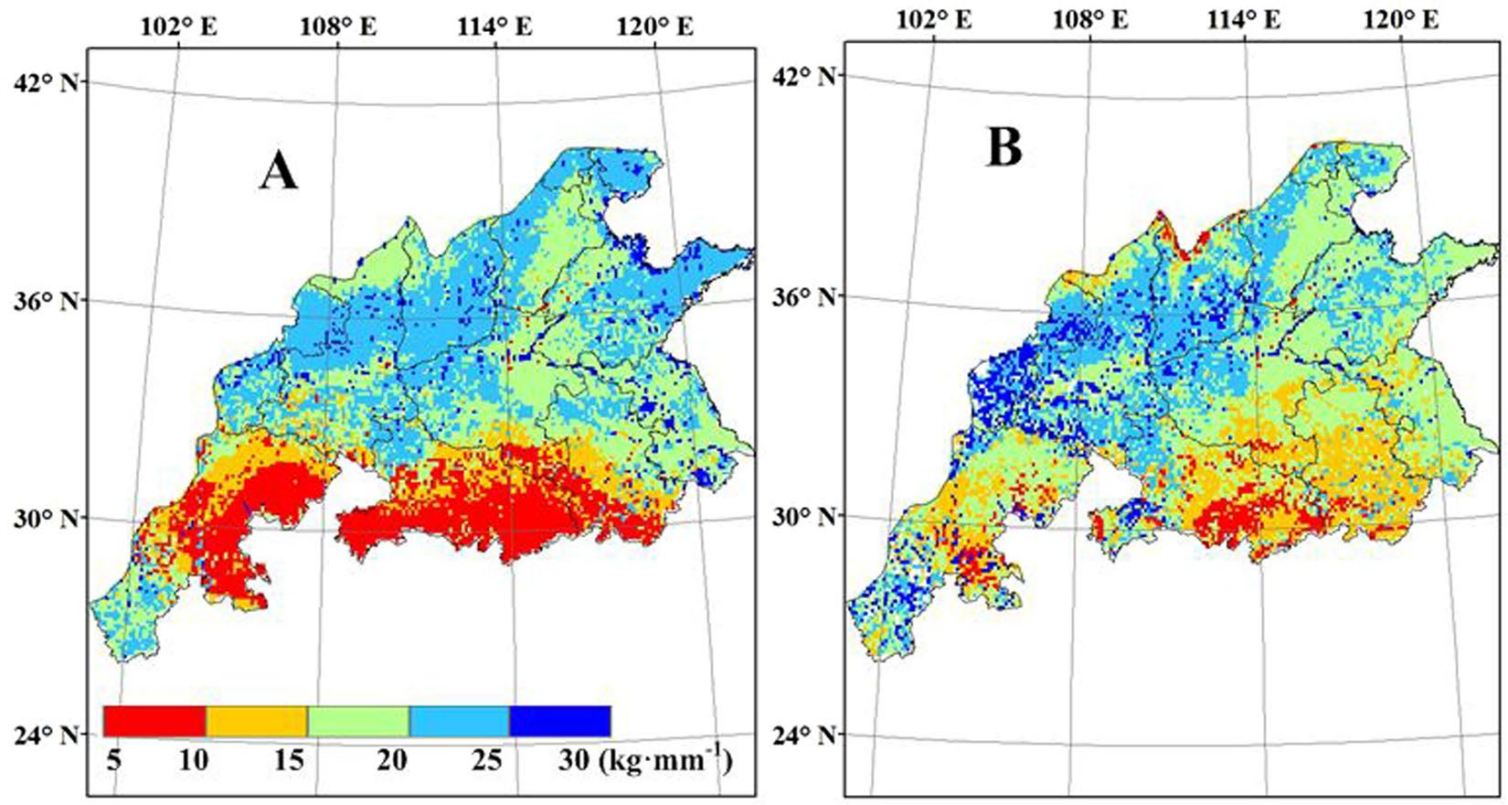
Average water limiting index (WLI) from 1998 to 2008 in the main winter wheat production regions of China, (**A**) CERES-Wheat; (**B**) WheatGrow (This figure was created by ArcGIS 9.3, http://www.esri.com/arcgis/).

The spatial patterns of WLI values simulated by WheatGrow agree well with those simulated by CERES-Wheat (Fig. 11). The WLI values were used to identify yield-increasing potential if suitable water supplies are available. The WLI values varied from 40 to 9875 kg ha^−1^, with the highest values in the northern provinces of the main production region and the lowest values at Sichuan and Hubei provinces. Water was the largest limiting factor in Shanxi, Gansu and Shaanxi provinces, while water was not the main limiting factor in Sichuan and Hubei provinces and yield loss by water deficit accounted for 5–15% of all yield loss from factors such as water deficit, nutrient deficit, insect pests, diseases and weed competition. The WLI decreases from north to south, mainly due to lower precipitation in the northern regions. Along the same latitude, the WLI generally increases from west to east, due to lower precipitation in the western regions. Yield-increasing potential was greater in the northern area of the main wheat production regions, where the WLI was high (more than 4800 kg ha^−1^) if irrigation water was available. Southern areas such as Sichuan and Hubei provinces had higher cumulative P/PET (cumulative P/PET in the wheat growing period was over 4.3), and the rainfed yield was very close to potential yield. Therefore, the possibilities to increase yields by improving water supply strategies are limited in southern areas. These results reveal that there are negative linear relationships between the WLI and accumulated P/PET, with correlation coefficients of −0.59 and −0.80 for WheatGrow and CERES-Wheat, respectively.

### Average irrigation and IWUE

**Figure 12:**
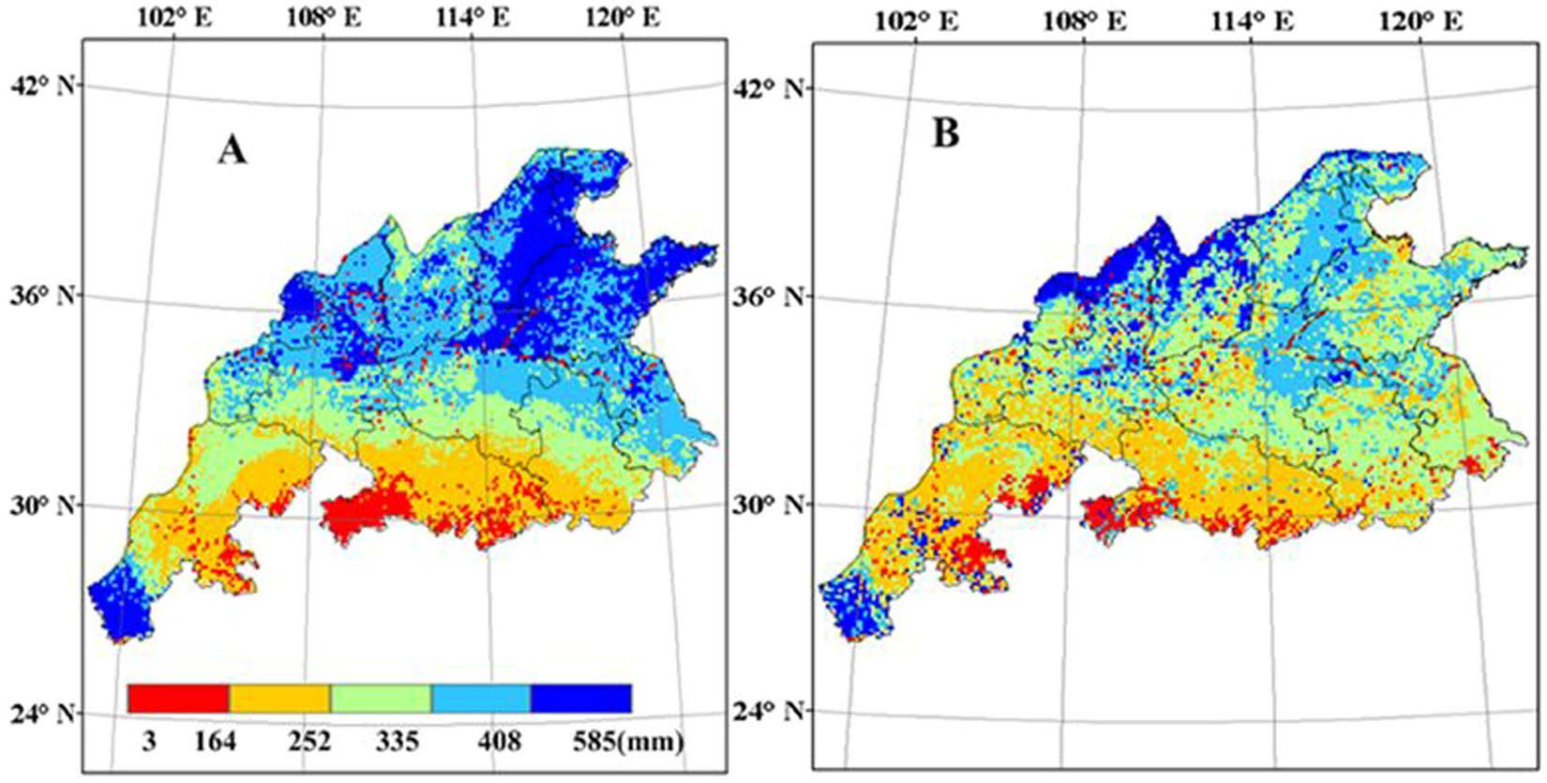
The average irrigation amount of winter wheat from 1998 to 2008 in the main production regions of China, (**A**) CERES-Wheat (**B**); WheatGrow (This figure was created by ArcGIS 9.3, http://www.esri.com/arcgis/).

During the simulation, irrigation was applied when soil moisture was less than 65% of field capacity. The average irrigation amount was the average water demanded for wheat to reach the potential yield. The spatial patterns of the average irrigation amounts simulated by WheatGrow agree well with those simulated by CERES-Wheat from 1998 to 2008, and these amounts decrease from north to south (Fig. 12). The average irrigation amount was greater than 335 mm for CERES-Wheat in the most regions in Shandong, Hebei, Shanxi and Shaanxi (light blue and dark blue in Fig. 12), while the average irrigation amount is greater than 300 mm for the WheatGrow model. In the southern areas, there are obvious changes in dry/wet status in the growing season, implying seasonal drought during wheat production. The results display good linear relationships between the average irrigation amount and the yield gaps between potential and rainfed yield, with correlation coefficients of 0.57 and 0.77 for WheatGrow and CERES-Wheat, respectively.

**Figure 13:**
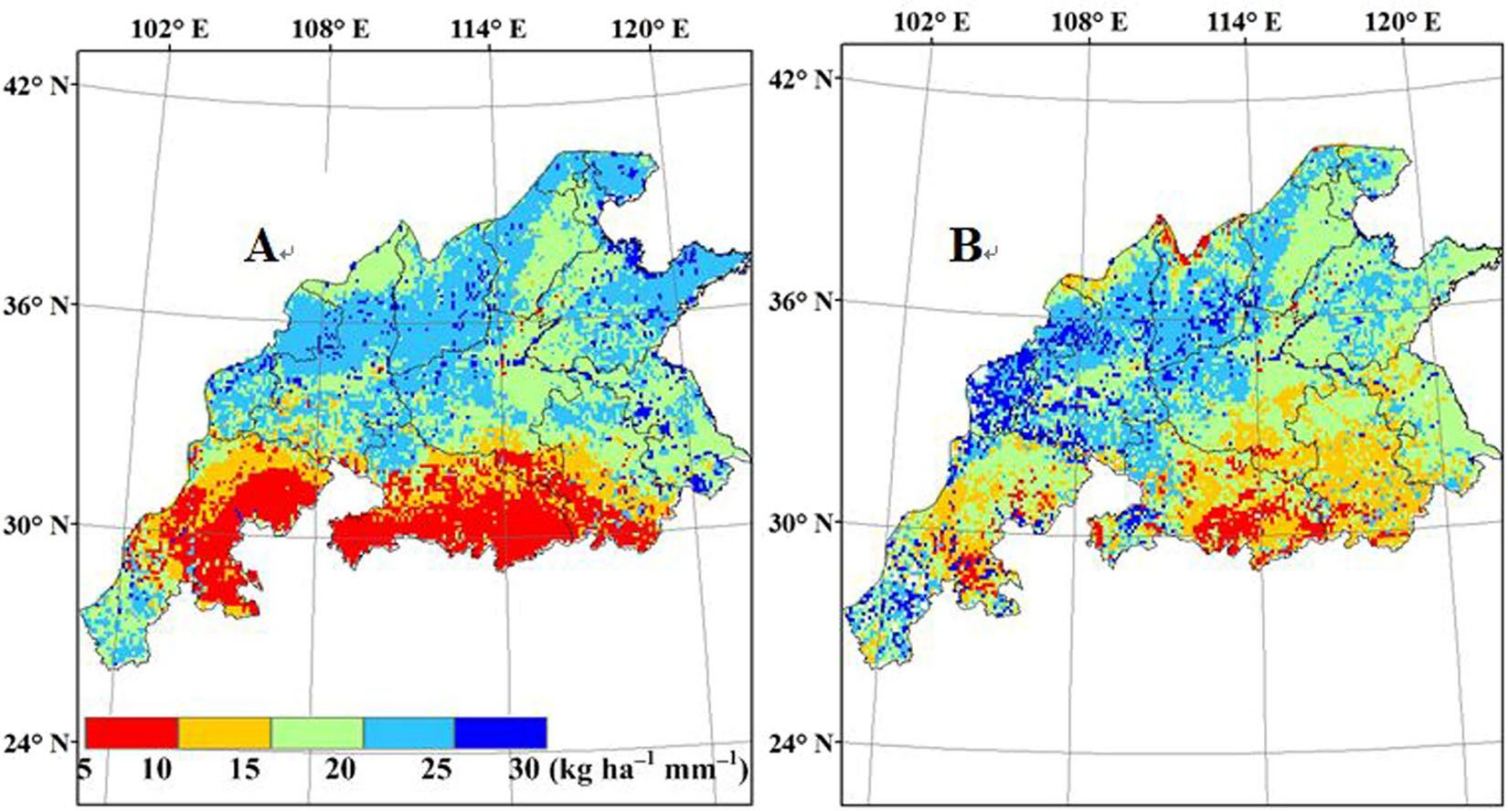
Simulated wheat irrigation water use efficiency (kg ha^−1^ mm^−1^) from 1998 to 2008 in the main production regions of China, (**A**) CERES-Wheat; (**B**) WheatGrow (This figure was created by ArcGIS 9.3, http://www.esri.com/arcgis/).

The spatial distribution map of wheat irrigation water use efficiency (IWUE) is shown in Fig. 13. Relatively high IWUE was found in western Shandong and southern Sichuan, as well as in northern Henan, Shanxi and Shaanxi. The IWUE are greater than 20 kg ha^−1^ mm^−1^ in most parts of those areas. Rainfall basically meets the water demand for wheat growth in most regions of southern China. There were no significant differences in wheat yield between rain-fed and full-irrigation conditions. Therefore, the IWUE was close to zero. As a result, full use should be made of the limited water supply in the regions with relatively high IWUE.

### Uncertainty analysis

**Figure 14:**
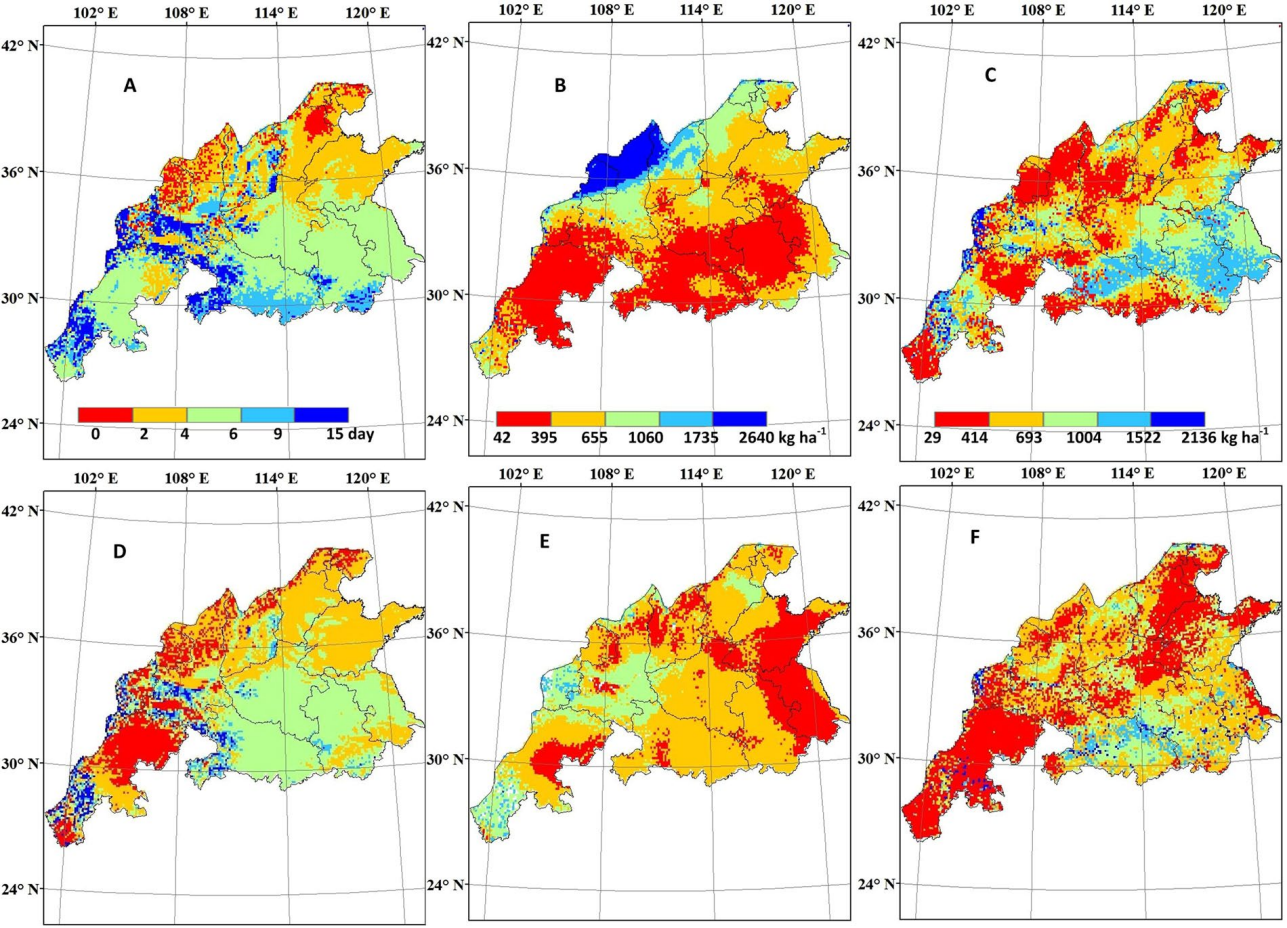
Spatial patterns of RMSD for maturity date (**A**,**D**), potential yield (**B**,**E**) and rainfed yield (**C**,**F**) of winter wheat, (**A**–**C**) CERES-Wheat; (**D**–**F**) WheatGrow (This figure was created by ArcGIS 9.3, http://www.esri.com/arcgis/).

The RMSD in Fig. 14 quantified the estimated uncertainty in the maturity date, potential yield and rainfed yield from 1998 to 2008. The RMSD values for maturity dates in high-altitude regions were higher than those in the other regions. The uncertainty estimated by CERES-Wheat for maturity date in the northern provinces was higher than that in the southern provinces. The RMSD values for potential yield simulated by the CERES-Wheat model in the northern part of the wheat production region were higher than those in the other regions. Most of the RMSD values for rainfed yield simulated by the CERES-Wheat model were greater than 1000 kg ha^−1^ in Jiangsu and Anhui provinces. The uncertainties estimated by the WheatGrow model in Hubei province were higher than those in the other provinces.

## Discussion

Compared with other studies about yield gaps, we adopted some new techniques to simulate regional yield. First, we used thin-plate smoothing splines in the ANUSPLIN software to generate daily meteorological data. Based on the thin-plate smoothing spline function, ANUSPLIN can develop accurate interpolation and reflect the relationships between the meteorology variables and their influenced factors, and the software can especially adapt to time series of data^41^. For this research area, a comparative study was done in our lab to evaluate the methods of inverse distance weighting, co-kriging, and thin plate spline for interpolating the average meteorological element (including maximum air temperature, minimum air temperature, hours of sunshine, and precipitation) of the 15th of each month from the 1951–2005 comprehensive observation data of 559 meteorological stations in China. The thin plate spline interpolation method demonstrated the optimal spatial interpolation for interpolating and rasterizing the daily meteorological elements in China^34^. Second, we estimated ecotype cultivar parameters by using an MCMC method that is extensively validated at the regional scale for the WheatGrow^56^ and CERES-Wheat models^61^. Bayesian methods can greatly improve the accuracy of model predictions. Compared to the Generalized Likelihood Uncertainty Estimation method (GLUE)^63^, which is used to estimate parameters for DSSAT model, the values of mean squared error from model predictions are lower with the MCMC method than with the GLUE method^64^. Third, soil moisture was obtained from China Land Soil Moisture Data Assimilation System (CLSMDAS)^19^ which based on remote sense data and land process models. The assimilated high-quality soil moisture grid-point data provide important basic information for initial soil water content. The soil moisture content is important for predicting crop productivity and water use under insufficient irrigation conditions^65^. Compared with previous studies, these three new techniques made our studies more accurate for predicting regional wheat productivity.

The spline interpolation method was also used to interpolate the surface of actual yields. 15% of the actual yield variability cannot be explained by splines interpolation. First, wheat yield variability was caused by diseases, pests and weeds in some regions, which cannot be explained by the interpolation method. Second, any interpolation method cannot explain the whole yield variability. Because of the smoothing effect of interpolation method, the variation of the estimated values is less than that of the actual values^66^. Third, relatively sparse eco-sites in some areas also affected the accuracy of interpolation.

Potential production (Fig. 7) assumes that nutrients and water are in adequate supply while weed, pest and disease control and other crop management practices are optimal to simulate yield levels. In the actual production, efforts are directed towards breaking the upper limit of wheat yield by improving production conditions. Wheat yield records exist in several provinces, such as 11519.7 kg ha^−1^ in Shandong province, 11322 kg ha^−1^ in Henan province, 10528.5 kg ha^−1^ in Hebei province, 10288.5 kg ha^−1^ in Shanxi province and 10451.1 kg ha^−1^ in Jiangsu province^67^. The present study has exhibited great regional differences in climate, cultivar, soil and management, causing marked spatial variation in wheat yields. There is good consistency between the yield records and the simulated results in this study.

Several crop model studies^68, 69^ demonstrated that the average potential yields of winter wheat in the North China Plain (NCP) ranged from 5900 kg ha^−1^ to 17400 kg ha^−1^. Lu and Fan^25^ showed that average potential yield showed an increasing trend from north to south in most of the NCP by using the EPIC (Environment Policy Integrated Climate) model. In our research, Shandong province has the highest potential yield. Li^69^ used the APSIM model to predict the wheat productivity of the NCP, which includes Shandong, Henan and Hebei provinces. Spatial distributions of potential yield and yield gap were consistent with those in our research. However, the potential yield in our research is higher than that simulated by the APSIM model in above mentioned provinces. Yg percentages in those three provinces are greater than 20% in our research, while the Yg percentage simulated by the APSIM model in the centre of Henan province is less than 20%^69^.

The accumulated precipitation often contains some water not used for wheat growth and is affected by the length of growing season. Compared with accumulated precipitation during the growing season, accumulated P/PET could well reflect a dry/wet status in the wheat growing period^2^. A clear correlation was found between maize yield and P/PET. In the northern part of China, precipitation is the limiting factor for winter wheat yield, and an adequate water supply is important for yield enhancement^21^. The analysis of WLI can be used to identify where yields can be increased with sufficient irrigation. In the southern region of China radiation and temperature are major limiting factors^21^. Excessive soil moisture due to soils with high clay contents is another reason for wheat yield reduction. Too much rain in the spring often creates excessive accumulated water in the soil, and the physiological functions of the root system could be inhibited^49^. Waterlogging stress and drought could both strongly reduce the total biomass and change the dry matter partitioning between above and below ground components^50^.

Based on crop-water relations, WheatGrow quantified water stress factors for the simulation of crop growth, integrating the availability of soil moisture, the sensitivity of crop development stages and different physiological processes (such as photosynthesis, transpiration and partitioning) to drought and waterlogging stress, and the effect of waterlogging duration^49, 50^. CERES-Wheat does not consider the negative effects caused by waterlogging on wheat growth. Xiong^18^ evaluated wheat production in the southern areas of China using CERES-Wheat, and the model failed to produce good results, possibly due to the inability of the model to simulate waterlogging effects (which are common in this area). In this study, the yield gap between potential and rainfed yields simulated by WheatGrow is higher than that simulated with the CERES-Wheat, also likely due to the difference in waterlogging simulation in two models^49^. In addition, the relatively higher soil clay contents can easily result in the generation of waterlogging in the southern areas^50^. The potential yield simulated by the CERES-Wheat model is lower than that of WheatGrow in the northern region of Shanxi and Shaanxi provinces, because the WheatGrow model does not consider the effects of freezing on crops. Therefore, low temperature may reduce the wheat yield in the northern part of the main wheat production regions of China, but the WheatGrow model may overestimate the yield of these regions.

In Luancheng in Hebei province, Sun^60^ showed that the water deficit for wheat reached almost 290–300 mm in wet and normal years and 350 mm in dry years. The average irrigation corresponding to the longitude and latitude of Luancheng for WheatGrow and CERES-Wheat were 317.4 mm and 396.3 mm, respectively, in the present study. The use of groundwater for irrigation is required to maintain the current wheat yield levels. However, the extensive use of groundwater for irrigation and increased industrial water use has caused a rapid decline in the groundwater table, which will lead to unsustainable agricultural production^39^. For long-term sustainability, use of groundwater in those areas must be reduced, while the current wheat yield level should be maintained. Therefore, improving the efficiency of existing water resources and developing drought-tolerant wheat varieties are the most important strategies in the northern areas, as proposed by Wang^70^. In the southern region of China, most areas are humid, with abundant precipitation. The seasonal distribution of precipitation in this region is uneven. Therefore, seasonal waterlogging and drought can coexist in these areas^53^. Rational distribution of water during the wheat growing period is the most important strategy for improving wheat yield in the southern region.

The estimated uncertainties at the regional scale were quantified using RMSD. The RMSD values for maturity date in the high-altitude regions were larger than those in other regions, because the temperature in high-altitude regions fluctuated greatly between years. The RMSD values for potential yield simulated by the CERES-Wheat model in the northern part of the wheat production regions were higher than those in the other regions. Compared with the WheatGrow model, the CERES-Wheat model considered the effect of freezing on wheat. Therefore, low temperature may reduce potential yield in some years in the northern part of the wheat production regions, which will cause great uncertainty in these regions. The RMSD values for rainfed yield in Jiangsu, Anhui and Hubei provinces are larger than other provinces, which can be ascribed to the asymmetrical distribution of precipitation between years. The RMSD values for rainfed yield simulated by the WheatGrow model in the southern part of Hubei province were larger than those from the CERES-Wheat model, because the WheatGrow model considers the negative effects of waterlogging on wheat growth. Therefore, waterlogging may reduce rainfed yield in some years in the southern part of Hubei province, which will cause great uncertainty in this regions.

In the future, investigations should be made to minimize the gap between the simulated and observed yields under different production conditions with these two models. Meanwhile, the regionalization methods should be optimized for spatial inputs of weather, cultivar, soil and management in model simulations. Two models underpredicted yields in highly productive regions of Eastern China and overpredicted yields in less productive regions of Western China. Balkovic met the same problem in regional crop yield validation in Europe^71^. Additional effort is necessary for improving accuracy in predicting regional potential wheat productivity using growth models.

## Conclusion

This study estimated the regional productivities of winter wheat at different levels in the main production regions of China by integrating GIS with two wheat simulation models: WheatGrow and CERES-Wheat. The results showed that the spatial distribution patterns predicted by the two wheat models were similar for the maturity date, potential yield, rainfed yield, yield gap between potential yield and actual yield, and water limitation index and irrigation water use efficiency. The spatial distribution of potential wheat yield was consistent with the accumulated hours of sunshine. The rainfed yield roughly followed the pattern of the ratio of accumulated precipitation to accumulated potential evapotranspiration (P/PET). The gaps between the potential and the actual yield vary from 382 to 7515 kg ha^−1^, with the highest values in Shanxi, Gansu and Shaanxi provinces and the lowest values in Sichuan province. Through further analyses, the limiting factors for regional wheat productivity were revealed, and effective strategies were suggested to enhance the potential regional productivity. Improving water resource use efficiency and cultivating drought-tolerant wheat varieties are the most important strategies in Shandong, Hebei, Shanxi and Shaanxi provinces, while rational distribution of water is urgently needed in Sichuan and Hubei provinces. These results can provide information for national policy making and water redistribution in the wheat production areas of China.
